# Genetic profiles of cervical tumors by high-throughput sequencing for personalized medical care

**DOI:** 10.1002/cam4.492

**Published:** 2015-07-08

**Authors:** Etienne Muller, Baptiste Brault, Allyson Holmes, Angelina Legros, Emmanuelle Jeannot, Maura Campitelli, Antoine Rousselin, Nicolas Goardon, Thierry Frébourg, Sophie Krieger, Hubert Crouet, Alain Nicolas, Xavier Sastre, Dominique Vaur, Laurent Castéra

**Affiliations:** 1 Department of Cancer Biology and Genetics, CCC François BaclesseCaen, France; 2 Inserm U1079Rouen, France; 3 Recombination and Genetic Instability, UMR 3244, Institut CurieParis, France; 4 Biopathology Department, Institut CurieParis, France; 5 Department of Radiotherapy, Institut CurieParis, France; 6 Department of Genetics, University HospitalRouen, France; 7 Caen UniversityCaen, France; 8 Gynecology Oncology Department, CCC François BaclesseCaen, France

**Keywords:** Cervix, diagnosis, NGS, panel, targeted therapie

## Abstract

Cancer treatment is facing major evolution since the advent of targeted therapies. Building genetic profiles could predict sensitivity or resistance to these therapies and highlight disease-specific abnormalities, supporting personalized patient care. In the context of biomedical research and clinical diagnosis, our laboratory has developed an oncogenic panel comprised of 226 genes and a dedicated bioinformatic pipeline to explore somatic mutations in cervical carcinomas, using high-throughput sequencing. Twenty-nine tumors were sequenced for exons within 226 genes. The automated pipeline used includes a database and a filtration system dedicated to identifying mutations of interest and excluding false positive and germline mutations. One-hundred and seventy-six total mutational events were found among the 29 tumors. Our cervical tumor mutational landscape shows that most mutations are found in *PIK3CA* (E545K, E542K) and *KRAS* (G12D, G13D) and others in *FBXW7* (R465C, R505G, R479Q). Mutations have also been found in *ALK* (V1149L, A1266T) and *EGFR* (T259M). These results showed that 48% of patients display at least one deleterious mutation in genes that have been already targeted by the Food and Drug Administration approved therapies. Considering deleterious mutations, 59% of patients could be eligible for clinical trials. Sequencing hundreds of genes in a clinical context has become feasible, in terms of time and cost. In the near future, such an analysis could be a part of a battery of examinations along the diagnosis and treatment of cancer, helping to detect sensitivity or resistance to targeted therapies and allow advancements towards personalized oncology.

## Introduction

Cancer treatment faces a major evolution since the advent of targeted therapies. It is now possible to specifically treat tumors presenting with a clearly identified genetic alteration(s). It is the case of nonsmall-cell lung cancers associated with *EGFR* mutations that responds to specific tyrosine kinase inhibitors 1. This type of therapy can only be effective in the case of a known molecular target. It requires the characterization of various alterations (commonly called actionable mutations) that a tumor may accumulate. Establishment of such genetic profiles would allow sensitivity, resistance, and toxicity predictions for such therapies 2. Sequences from many human genomes have shown a wide interindividual genetic heterogeneity 3. Variations among individuals are attributed to “germline” mutations and constitute the individual's inherited genetic characteristics. These mutations, however, should be distinguished from other mutations that may occur during the individual's life, and also, might only be observed in one organ or some tissue. These events are called somatic mutations and have been shown to provoke various oncogenic processes 2 (they are called “driver” mutations).

Many research teams, brought together in international consortiums, are engaged in the characterization of mutations causing tumorigenesis. The Catalog Of Somatic Mutations In Cancer (COSMIC) Project 4, for example, aims at creating a database gathering all somatic mutations already described. The International Cancer Genome Consortium plans to build a comprehensive catalog of somatic mutations in tumors of 50 different types/subtypes of cancer, with additional epigenomic and transcriptomic information, with the aim of highlighting differences and common abnormalities across tumor types 5. These works on tumor genetic profiling have demonstrated that tumors of different types can share protumorigenic signaling pathways, defined by common driver mutations which could also be actionable 6. Consequently, it makes sense to explore the presence or absence of mutations within these driver genes, to identify biomarkers for sensibility or resistance to treatments, and to indicate those patients most likely to benefit from targeted therapies.

The number of genes to test, in order to establish a cancer gene map, is huge 7. This approach is made possible by a new generation of sequencing devices (Next Generation Sequencing, NGS), able to analyze the equivalent of several entire genomes 8. However, sequencing a whole genome can be quite long, regardless of analysis time for such an extensive quantity of data. Another approach consists of limiting the analysis to exomes or several hundreds of genes, using targeted methods. Using a rational approach, which targets a panel consisting of a few hundred genes, considered to be “actionable” and/or “driver”, it is possible to sequence and characterize a tumor, depending on its major molecular characteristics. The main advantages of this approach lie in the reduced delay for reporting the results and limited costs, compatible with a diagnostic use, while maintaining sufficient sequencing quality to detect somatic variations 9.

The sensitivity of these methods depends on bioinformatic tools specifically developed to highlight somatic mutations in cancer, by comparing healthy tissue and tumor DNA from the same individual (paired-mode). These tools verify that a somatic mutation found in tumor does not actually correspond to germline mutation or systematic false-positive mutation, found in healthy tissues 10. In some cases, it is difficult to obtain the matched healthy tissue in a clinical context, depending on legislative (local or national), ethical or logistical considerations; a constraint that must be overcome. On the other hand, the detection of mutations strongly depends on algorithms and their adjusted parameters used. In addition, their sensitivity depends on the type and rate of mutation present in the tumor. To date, there is no global consensus for the use of a particular algorithm or mode of analysis 10.

We report on the genetic profiles of cervix uteri tumors which represents the fourth leading cause of death by cancer in females worldwide 11. It is well-known that some human papillomavirus (HPV) types are the cause of this cancer and its development is linked to the stable insertion of the HPV genome into the tumor's DNA 12. Cancer of cervix uteri can go undetectable for years and years, and is associated with a 5-year survival rate of 68% 13, depending on clinical stage at presentation: the 5-year relative survival rate is 91%, 58%, and 17% for patients with localized, regional, and metastatic disease, respectively (http://seer.cancer.gov/statfacts/html/cervix.html). Moreover, up to one-third of patients will develop recurrent tumors mostly within 2 years after initial treatments 14. Most recurrences being detected by imaging or medical examination, it is essential that highly specific, sensitive and less invasive markers are identified to predict response to treatment, disease progression, and to develop personalized therapies.

In our laboratory, we have developed the sequencing of a panel of genes associated with tumors of cervix uteri, including exons within 226 genes chosen for their actionable nature and their potential implication in cervical tumorigenesis (Table1). To overcome the lack of matched healthy tissue, an average healthy tissue has been generated in silico from nonmatched healthy tissues at our disposal. The aim of the study was to prove that a tumor can be managed in a clinical context using NGS technology and automated bioinformatic pipeline and to identify potential actionable mutations that could have a direct link with treatments.

**Table 1:** Genes selected for cervix uteri cancer panel

*ABL1*	*CDK8*	*FGFR3*	*LTK*	*NSD1*	*SMAD2*
*ABL2*	*CDKN1A*	*FGFR4*	*MAP2K1*	*NTRK1*	*SMAD3*
*AFF3*	*CDKN2A*	*FHIT*	*MAP2K2*	*NTRK2*	*SMAD4*
*AKT1*	*CEACAM5*	*FKBP9*	*MAP2K4*	*NTRK3*	*SMARCA4*
*AKT2*	*CEBPA*	*FLT1*	*MCL1*	*PAK3*	*SMARCB1*
*AKT3*	*CHEK1*	*FLT3*	*MDM2*	*PARP1*	*SMO*
*ALK*	*CHEK2*	*FLT4*	*MDM4*	*PAX5*	*SOCS1*
*APC*	*CREBBP*	*FOXP4*	*MED1*	*PDGFRA*	*SOX10*
*AR*	*CRKL*	*GATA1*	*MEN1*	*PDGFRB*	*SOX2*
*ARFRP1*	*CROCC*	*GLIS2*	*MET*	*PDPK1*	*SRC*
*ARID4A*	*CSF1R*	*GNA11*	*MGMT*	*PIK3CA*	*STK11*
*ATM*	*CSMD1*	*GNAQ*	*MITF*	*PIK3R1*	*SUFU*
*AURKA*	*CTLA4*	*GNAS*	*MLH1*	*PKHD1*	*TBX22*
*AURKB*	*CTNNB1*	*GPR124*	*MLL*	*PLCG1*	*TCF4*
*BCL2*	*CTTN*	*GUCY1A2*	*MLL2*	*PLEKHO2*	*TENM1*
*BCL2A1*	*DAPK1*	*HIC1*	*MLL3*	*PRDM9*	*TERT*
*BCL2L1*	*DDX3X*	*HNF1A*	*MPL*	*PRKDC*	*TET2*
*BCL2L2*	*E2F3*	*HOXA3*	*MRE11A*	*PTCH1*	*TGFBR2*
*BCL6*	*EGFR*	*HRAS*	*MSH2*	*PTEN*	*TIMP2*
*BRAF*	*EMSY*	*HSP90AA1*	*MSH6*	*PTK2*	*TIMP3*
*BRCA1*	*EPHA3*	*IDH1*	*MTOR*	*PTK2B*	*TNFAIP3*
*BRCA2*	*EPHA5*	*IDH2*	*MUC1*	*PTPN11*	*TNFRSF10C*
*CADM1*	*EPHA6*	*IGF1R*	*MYB*	*PTPRD*	*TNFRSF10D*
*CASP8*	*EPHA7*	*IGF2R*	*MYC*	*RAD51*	*TOP1*
*CASZ1*	*EPHB1*	*IKBKE*	*MYCL1*	*RAD51B*	*TP53*
*CAV1*	*EPHB4*	*IKZF1*	*MYCN*	*RAF1*	*TP63*
*CBL*	*EPHB6*	*INHBA*	*MYH15*	*RARA*	*TP73*
*CCND1*	*ERBB2*	*IRF6*	*NF1*	*RARB*	*TSC1*
*CCND2*	*ERBB3*	*IRS2*	*NF2*	*RASSF1*	*TSC2*
*CCNE1*	*ERBB4*	*JAK2*	*NFE2L2*	*RB1*	*TSHR*
*CCNL1*	*ESR1*	*JAK3*	*NFIB*	*REL*	*USP9X*
*CDC73*	*EZH2*	*KDR*	*NKX2-1*	*RET*	*VHL*
*CDH1*	*FAM123B*	*KEAP1*	*NOTCH1*	*RICTOR*	*WT1*
*CDH2*	*FANCC*	*KIAA0774*	*NOTCH2*	*ROS1*	*ZBTB7C*
*CDH20*	*FANCF*	*KIT*	*NOTCH3*	*RPS6KB1*	*ZNF668*
*CDH5*	*FBXW7*	*KLF5*	*NOTCH4*	*RPTOR*	*ZNF91*
*CDK4*	*FGFR1*	*KRAS*	*NPM1*	*RUNX1*	
*CDK6*	*FGFR2*	*LRP1B*	*NRAS*	*RUNX1T1*	

## Material and Methods

### Tumor samples

Twenty-five squamous cell carcinoma and four adenocarcinoma tumor samples were obtained under approved protocols from the Curie Institute in Paris, France (Table2). Seven healthy tissue samples were obtained from cervical surgical specimen provided by the Anatomic Pathology laboratory of cancer center François Baclesse in Caen, France. All patients gave written informed consent before entering the study.

**Table 2:** Demographic, histological, and biological characteristics

Patient no.	Histological type	Tumor stage (FIGO)	Age at diagnosis	HPV type
1	SCC	IIB	40	HPV 18
2	SCC	IB	33	HPV 18
3	Adenocarcinoma	II	45	HPV 18
4	SCC	IB	37	HPV 18
5	SCC	IB	34	HPV 16
6	SCC	IB2	47	HPV 16
7	Adenocarcinoma	IIB	49	HPV 16
8	SCC	IVB	57	HPV 16
9	SCC	IB1	33	HPV 16
10	SCC	NA	NA	HPV45
11	SCC	IIB	60	HPV 16
12	SCC	IIB	42	HPV 18
13	SCC	IIB	47	HPV 16, 18
14	SCC	IIB	34	HPV 16
15	SCC	IIB	68	HPV 16
16	SCC	IIB	43	HPV 18
17	SCC	IIB	44	HPV 51
18	SCC	IIB	65	HPV 33
19	SCC	IIB	45	HPV 73
20	SCC	IIIB	43	HPV 16
21	SCC	IIB	53	HPV 16
22	Adenocarcinoma	IB2	42	HPV 16
23	Adenocarcinoma	IIB	54	HPV-
24	SCC	IIB	54	HPV 18
25	SCC	III	33	HPV 16
26	SCC	IIB	55	HPV 16
27	SCC	IV + metastasis	44	HPV 73
28	SCC	IB1	25	HPV 18
29	SCC	IB2	31	HPV-

FIGO, International Federation of Gynecology; HPV, human papillomavirus; SCC, squamous cell carcinoma; NA, not available.

### Sample preparation and next-generation sequencing

Sequencing methods were described previously 15, enabling analysis of two tumors and one healthy tissue sample in the same sequencing run. The SureSelect-targeted enrichment process (Agilent, Santa Clara, CA) was performed after combining the indexed samples, equimolarly. Libraries were then sequenced on a MiSeq (Illumina, San Diego, CA), using the paired-end 2 × 150 bp method (full protocol available on request).

### Bioinformatic analysis

The CASAVA suite v1.8 (Illumina) was used for demutiplexing and generating fastq files. These raw data were then analyzed by a home-made pipeline (BAPT, Bioinformatic Analysis Pipeline Toolkit), to automate alignment, variant-calling, and annotation steps. Reads were first mapped to a reference genome (hg19) using BWA algorithm 16 and output files were reworked following GATK 17 (Genome Analysis ToolKit, Broad Institute) best practices, especially to calculate quality scores and undergo local realignments. Five variant-callers were used to call single-nucleotide substitutions and insertion–deletion (indels) events: HaplotypeCaller 18, UnifiedGenotyper 18, LofreqStar 19, Varscan2 20, and MuTect 21. The BAM files from seven healthy samples were used by randomly selecting reads from each file, to create an average healthy synthetic file (using SAMtools 22). This “synthetic” BAM file was used in paired-mode during the variant-calling step with the programs LofreqStar, MuTect, and VarScan2. All variants found by the variant callers were annotated with Alamut-Batch (Interactive Biosoftware, Rouen, France) and integrated into a database called CanDiD (Cancer Diagnostic Database, PostgreSQL) (Fig. S1). Variants were retrieved from CanDiD database according to five criteria: (1) the variant was found on a list of preferred transcripts; (2) the variant was in targeted zones; (3) the variant was within the coding sequence or ±10 bp within the intronic sequences; (4) the variant was in a canonical splicing mutation sites or when outside they induced a 15% decrease of MaxEntScan score and a 5% decrease of the SpliceSiteFinder score 23 (hereafter called splicing mutation); and (5) the variant was observed 10 times at most in the Exome Sequencing Project Database (ESP; http://evs.gs.washington.edu.EVS/). Identified variants passed through a home-made filtration system (scripted on Python programming language, available on request) which compares sequencing data from the five variant-callers and tumor samples versus healthy samples, in order to rule out false-positive and germline variants. Data from each variant were first compared to both tumor and healthy tissue samples, to evaluate whether the variant have a greater presence in the tumor than in the healthy samples. Variants were then filtered based on their quality score (the main criteria being the PHRED score and depth of coverage). The main objective was to obtain, for each tumor, a list of variants with a high probability of being somatic. Only variants seen by at least 2 variant callers were further considered.

Results were annotated and interpreted in sequence with: (1) two mutation impact prediction systems SIFT 24, Polyphen 25, (2) InterPro Database 26, (3) Clinical Trials.gov Database 27, (4) dbSNP 28, (5) COSMIC 4, (6) TARGET 29.

In the present study, all genes that constitute a target for the US Food and Drug Administration-approved targeted therapies were called actionable genes, regardless of the type of cancer. Similarly, a missense mutation was classified as deleterious if scored as “deleterious” by SIFT algorithm or “Probably Damaging” by Polyphen algorithm. Inactivating mutations (PTC, premature codon termination and splicing mutations) were directly considered as deleterious.

## Results

**Figure 1:**
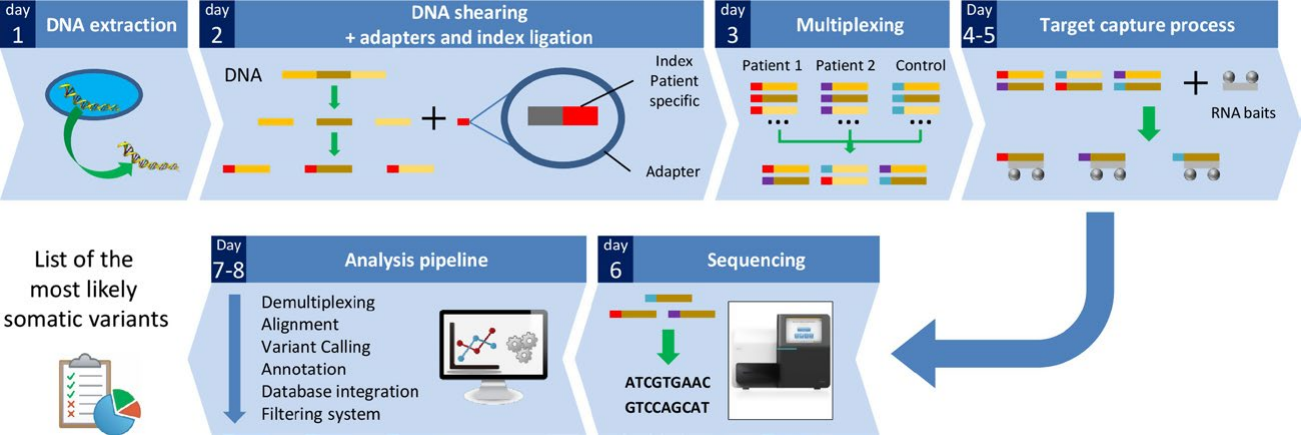
Sequencing workflow's major steps. After DNA extraction (Day 1), DNA molecules are sheared by sonification, and DNA fragments are ligated to adapters containing a patient-specific index (Day 2). DNA from 2 patients and 1 control are pooled equimolarly together during the multiplexing step (Day 3). Regions of interest (exons from 226 genes) are retrieved by a targeted enrichment system with biotinylated baits (Day 4–5). Then DNA is sequenced in an Illumina MiSeq (Day 6) and sequencing raw data are processed by the bioinformatic pipeline (Day 7–8), to extract the most likely somatic variations.

**Figure 2:**
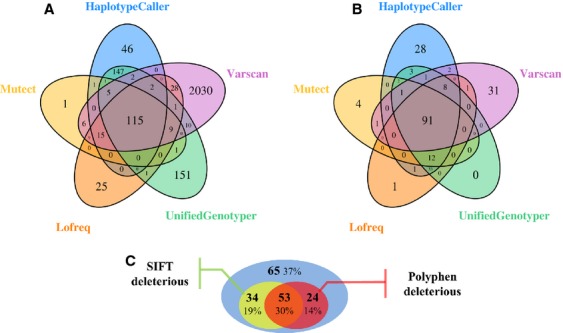
Representation of missense mutations found by each variant caller. (A) Mutations extracted directly from CANDID database (total: 2746). (B) mutations remaining after passing through filtration system (total: 221). (C) Proportion of mutations detected by at least 2 variant-callers classified as deleterious by SIFT or POLYPHEN.

Tumor samples from 29 patients with cervical cancer (stade I B to IV B), including 25 squamous cell carcinomas and 4 adenocarcinomas, have been sequenced (Table2), as well as seven samples from healthy cervical biopsy (“control” samples). The time from DNA extraction to acquisition of most likely somatic mutations was about height working days (Fig.1). The sequencing process produced an average of 8,078,023 reads per sample, with an average sequencing depth of 268× and >92% of nucleotides covered ≥50-fold. Consequently, only variants with an allele ratio of more than 5% were called. After data processing in the BAPT pipeline, 11,267 variants were included in the CanDiD database. After extraction from the database, 2746 missense mutations were collected from the various variant callers (Fig.2A). After passing through the filtration system, only 220 mutations were retained (Fig.2B; Table S2). Most of the mutations deleted by the filtration system were germline mutations detected by HaplotypeCaller or UnifiedGenotyper (from the control samples) or false-positive selected by Varscan due to its lack of specificity. Among all mutations found in the 29 tumor samples, 41% (91/220) were identified by all variant callers, assuring accuracy of the mutations detected. Only variants detected by two or more variant-callers were selected leading to 156 missense mutations selected, among which 29 are already referenced in the COSMIC database. Seven nonsense mutations, nine small insertion/deletions (indels) inducing PTC and four splicing mutations were also identified. Altogether, they represent an average of 6.1 mutations per tumor sample. For each patient, 75 percent of mutations were considered deleterious (by SIFT or POLYPHEN algorithm) (Fig.2C, Table S2).

No somatic missense mutation was found in the tumor suppressor gene *TP53*, which is consistent with molecular characteristics of cervix uteri tumors 30. Similarly, missense mutations were found in the oncogene *PIK3CA* (*n* = 8), the *KRAS* gene implicated into anti-EGFR therapies resistance (*n* = 4) and in the *FBXW7* gene, recently described in the cervix uteri tumors mutational landscape (*n* = 3) (Table S2). Other missense mutations were found in tumor suppressor genes, including the susceptibility gene for breast cancer *BRCA2* (*n* = 6) or the RAS family negative regulator *NF1* (*n* = 1). Twenty inactivating mutations (PTC and splicing mutations) were also identified in tumor suppressor genes including *RB1* (*n* = 1), *NF1* (*n* = 3) or *MLH1* (*n* = 2) (Table3).

**Table 3:** Inactivating mutations

Coding effect	Gene	Coding DNA sequence^1^	Protein sequence 1	Transcript	Patients tumor sample no.
Splicing mutation	*CBL*	c.1096−1G>A	p. ?	NM_005188	5
Potential splicing mutations	*mTOR*	c.2514+3G>C	p. ?	NM_004958	15
	*MYH15*	c.1993−14G>A	p. ?	NM_014981	14
	*NF1*	c.1392+5G>T	p. ?	NM_001042492	21
Nonsense mutations	*C11orf30*	c.3004C>T	p.Gln1002^*^	NM_020193	26
	*CEACAM5*	c.646G>T	p.Glu216^*^	NM_004363	26
	*EPHA5*	c.2458G>T	p.Gly820^*^	NM_004439	6
	*FBXW7*	c.1053G>A	p.Trp351^*^	NM_033632	26
	*MLH1*	c.1630C>Tc.755C>T	p.Gln544^*^p.Ser252^*^	NM_000249	20 5
	*RB1*	c.1399C>T	p.Arg467^*^	NM_000321	5
Frameshift mutations	*CASP8*	c.790dup	p.Val264Glyfs^*^13	NM_001080125	5
	*CHEK2*	c.1229del	p.Thr410Metfs^*^15	NM_001005735	11
	*CREBBP*	c.4477dup	p.Ile1493Asnfs^*^26	NM_004380	5
	*FGFR2*	c.962dup	p.Asn321Lysfs^*^21	NM_022970	5
	*FGFR4*	c.2396_2403del	p.Gly799Aspfs^*^12	NM_213647	7
	*NF1*	c.5907_5908delc.2033del	p.Arg1970Serfs^*^6p.Pro678Argfs^*^10	NM_001042492	5
	*NF2*	c.301del	p.Tyr101Ilefs^*^22	NM_181832	16
	*NOTCH2*	c.6909del	p.Ile2304Leufs^*^2	NM_024408	5

Mutations are classified by mutation type.

1 Nomenclature according HGVS guidelines (Human Genome Variation Society).

**Figure 3:**
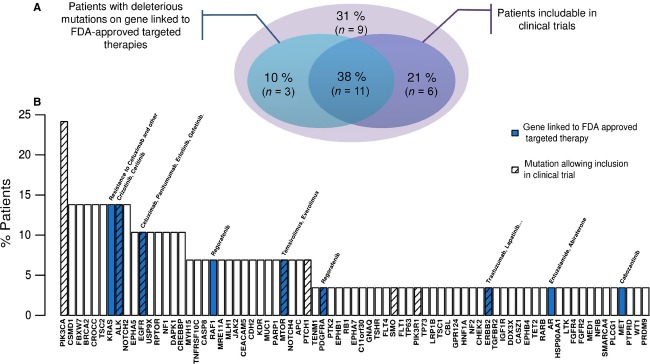
Distribution of deleterious mutations among the 29 tumors. A gene is considered actionable if linked to targeted therapies approved by Food and Drug Administration. A mutation is classified as deleterious if considered as such by SIFT algorithm or POLYPHEN algorithm. (A) Proportion of tumors with a deleterious mutation on gene considered actionable (blue) and proportion of patients for whom their deleterious mutations could allow inclusion in a clinical trial (purple). (B) Representation of most mutated genes across tumors.

Among other actionable genes, deleterious missense mutations were observed in *ALK* (*n* = 4), *AR* (*n* = 1), *EGFR* (*n* = 3), *ERBB2* (*n* = 1), *MET* (*n* = 1), *mTOR* (*n* = 2), *PDGFRA* (*n* = 1) and *RAF1* (*n* = 2) (Table4). Considering only deleterious mutations, 48% (*n* = 14/29) had at least one mutation in an actionable gene (Fig.3A and B).

**Table 4:** Deleterious mutations found in actionable genes

Gene	Drugs in relation with gene of interest	Mutation	Number of tumors	Transcript	Associated clinical trial
*ALK*	Crizotinib, Ceritinib	A1266TA1234VV1149LR1120Q	4	NM_004304	NCT01548144, NCT01744652
*AR*	Entuzalamide, Abiraterone	K809N	1	NM_000044	–
*EGFR*	Cetuximab, Panitumumab, Erlotinib, Gefetinib, Afatinib, Vandetanib	S511YT259MA611T	3	NM_005228	NCT00770263
*ERBB2*	Trastuzumab, Pertuzumab, Lapatinib	L696F	1	NM_001005862	NCT01953926
*KRAS*	Resistance to Cetuximab and others	G12DG13D	4	NM_033360	–
*MET*	Cabozantinib	L342F	1	NM_001127500	–
*mTOR*	Temsirolimus, Everolimus	M813I	1	NM_004958	–
*PDGFRA*	Regorafenib	P441L	1	NM_006206	NCT02029001
*RAF1*	Regorafenib	C96FQ255H	2	NM_002880	–

Genes are linked to targeted therapies already approved by Food and Drug Administration in at least one indication. All potential drug targets of each therapy are considered.

Several clinical trials (www.clinicaltrials.gov database) are looking for mutations in specific genes to evaluate sensitivity or resistance to approved, or in clinical trials, targeted therapies, among which *PIK3CA*,*ALK*,*EGFR*,*mTOR*,*PTCH1*, *SMO,* and *PIK3R1* are included (Table S2). Overall, 59% of patients (17/29) would be eligible for potential inclusion in a clinical trial (Fig.3A and B), based on carrier gene mutations and tumor origin.

## Discussion

The aim of the study was to demonstrate in a clinical context the feasibility of detecting somatic variations, in cervical cancers, using a targeted sequencing approach. The use of a “synthetic healthy tissue sample” (see above Material and Methods section) from the sequencing data of several unpaired healthy tissue samples allows the use of somatic variant-callers (which compares healthy tissues from tumors to extract somatic mutations) to detect poorly represented mutations. This approach, however, probably tolerates a few germline mutations which cannot be differentiated from somatic mutations. In our study, some mutations found in *BRCA2* (hereditary predisposition for breast and ovarian cancer) or in *MLH1* (hereditary predisposition to Lynch syndrome) genes through the genetic characterization of tumors could be germline (Table S2). The identification of germline mutations in genes in relation with mendelian diseases must be taken into account and the appropriate ethical issues should be considered, a subject still of debate. Tools and methods developed in our laboratory should not be considered as an automatic interpretation system; they should be considered as help to ease work discerning the most relevant mutations. For instance, with our data, the number of mutations to analyze drops from about 388 to 6 for one tumor, which represents a huge time-saving for biologists who interpret data in a clinical context. Moreover, analysis time (±8 working days) is consistent with clinical practice, and should improve patient healthcare in a comprehensive way.

No somatic alterations were detected in the tumor suppressor gene *TP53* which seems consistent throughout our model. In cervical carcinomas *TP53* inactivation is linked to virus integration, which results in the expression of the E6 oncoprotein, able to induce the TP53 degradation via the ubiquitin pathway 30. Previous studies have already identified genes frequently mutated in cervical carcinomas, such as *PIK3CA* 31, *KRAS* 32 (the most mutated genes in cervix uteri cancer according to COSMIC data) or *FBXW7* 33. *PIK3CA* E545K (*n* = 4) and E542K (*n* = 2) have strong oncogenic properties due to increased kinase activity 34, consequences in growth factor-independent cell proliferation, resistance to apoptosis, and increased invasion and cell migration. This gene represents a prime target for drug development, and even if there is no available approved treatment, several clinical trials are currently testing *PIK3CA* selective inhibitors (ClinicalTrials ID: NCT01708161; NCT01928459). Two major mutations were also found in *KRAS*, G12D (*n* = 1) and G13D (*n* = 3), well known in colorectal cancer to predict a resistance to *EGFR* antibodies 35. *FBXW7* is a gene recently described as mutated in cervical carcinoma 33 with two missense mutations already described, R505G and R456C. There was also a novel mutation at a position already found mutated, R479Q. All of these point mutations are located in the WD repeats domain. This domain is used to form a complex in charge of degrading several products of proto-oncogenes (including *MYC*, *CCNE1*, *NOTCH1,* and *JUN*) 36. We found a novel truncating mutation W351*; it occurs just upstream of the WD domain (uniProt ID: Q969H0). Therefore, pipeline implementation seems consistent, since key elements form cervical tumorigenesis were found.

Considering all of the actionable genes as defined before (Table4), deleterious mutations could influence sensitivity or resistance to treatments. *ALK* mutations V1149L and A1266T are located next to previously described mutations L1152R 37 and G1269A 38, which have been identified as resistance mutations to Crizotinib and other tyrosine kinase inhibitors, and could then have the same impact on treatment response. Another mutation (T259M) has been observed in a specific subpart of extracellular domain of *EGFR*, called the dimerization loop 39. This loop is essential for receptor dimerization and hence activation. A previous study showed that mutations in this subpart were able to increase receptor oncogenic activity 40. *MET* had also a new mutation at residue L342, which correspond to a specific region of SEMA domain, considered as MET ligand-binding domain 41 and useful to receptor dimerization and activation 42. Other mutations classified as deleterious were detected in *mTOR* (M813I, splice mutation c.2514+3G>C) and *PDGFRA* (P441L) but their real impact on protein functionality or the effectiveness of related targeted therapies is still unknown.

Eighteen deleterious mutations identified from our cases reside in actionable genes (Table3), although their impact is still unclear for many. Therefore, functional studies should be performed to identify potential drug sensitivities or resistances to these targets. Deleterious classification (SIFT and POLYPHEN algorithm) should be used with caution since, for instance, *PIK3CA* mutations E545K and E542K are classified as tolerated by SIFT despite the fact that they are known driver mutations.

Among driver genes investigated in our study, interesting mutations were found on *BRCA2*, with 2 variants of unknown signification, classified here as deleterious missense mutations (L2721R and A2770D) in the OB1 (oligonucleotide/oligosaccharide-binding 1) domain, which serves to bind single-strand DNA. Functional assays showed that mutations in this domain can be disease-causing 43. Another mutation (V2066I) was observed in BRC repeat domains, essential for binding to RAD51 protein 44. The new PARP inhibitor Olaparib, now available, could potentially be of interest in these BRCA mutated tumors. Unfortunately, for the moment this drug is mainly tested in breast and ovarian cancers. Altogether, the real new challenge for a tumor's genetic profiling is the correct interpretation of mutation.

Sixty-nine percent of patients could have had their treatment decision guided by the targeted sequencing of 226 genes, either by revealing sensitivity or resistance to available drugs or by directing patients toward treatment protocols or diagnostic procedures being tested (Fig.3A). Even though no therapeutic answer could be provided by our “oncopanel” for certain patients, they were all analyzed for HPV insertion status. The HPV insertion site constitutes another biomarker in *cis*, which can provide information for targeted therapies. HPV has therefore been found inserted into *MYC*, *KLF5*, *KLF12,* as well as *RB1*, and many other important and potentially actionable genes (A. Holmes, unpubl. data). Such tests might participate in the overall study of cancer biomarkers in both prognostic (assessing tumor outcomes and deciding whether or not to undergo treatment) and predictive (evaluating sensitivity and resistance to treatments to choose the most effective drug) fields 45. The outcome of such studies strives toward personalized oncology, which is to provide the most appropriate treatment to the patient with the correct dosage. The overall benefits first and foremost concern patients, who receive the most effective and least toxic treatments. Such an approach in patient care has economic advantages too. The correct administration of the adequate drug instead of using other ineffective and expensive therapies appears to be cost-effective. It has been calculated that US health-care system could save up to $740 million dollars by only treating patients with Cetuximab with a wild-type *KRAS* status 46. Other studies suggest that pharmacogenomics can be a good way to improve cost-effectiveness 47. However, more studies are needed in this field to assess the real economic impact of these therapies 48.

Interpretation of somatic mutations remains challenging because, except for a few mutations already described, the impact of each mutation requires functional studies. In some countries, the inability to obtain matched healthy tissue in a daily clinical practice makes detection of somatic mutations more difficult. On the other hand, sequencing a limited number of genes selected for their implication in tumor development is now feasible in a clinical setting (in term of costs and time), and the “oncopanel” approach allows a larger screening power for diagnosis or treatment orientation by identification of sensitivity or resistance to drugs.

## Supporting Information

**Figure S1.** Somatic mutations analysis pipeline: BAPT.

**Table S2.** Somatic mutations found across the 29 patients.
