# Supplementary material for: Genetic profiles of cervical tumors by high-throughput sequencing for personalized medical care
Source: Cancer Med. 2015 Jul 8;4(10):1484–93. doi: 10.1002/cam4.492 (PMC4618619; doi:10.1002/cam4.492)
Supplement: Supplementary file 1 [file cam40004-1484-sd1.pptx]

## Slide 1
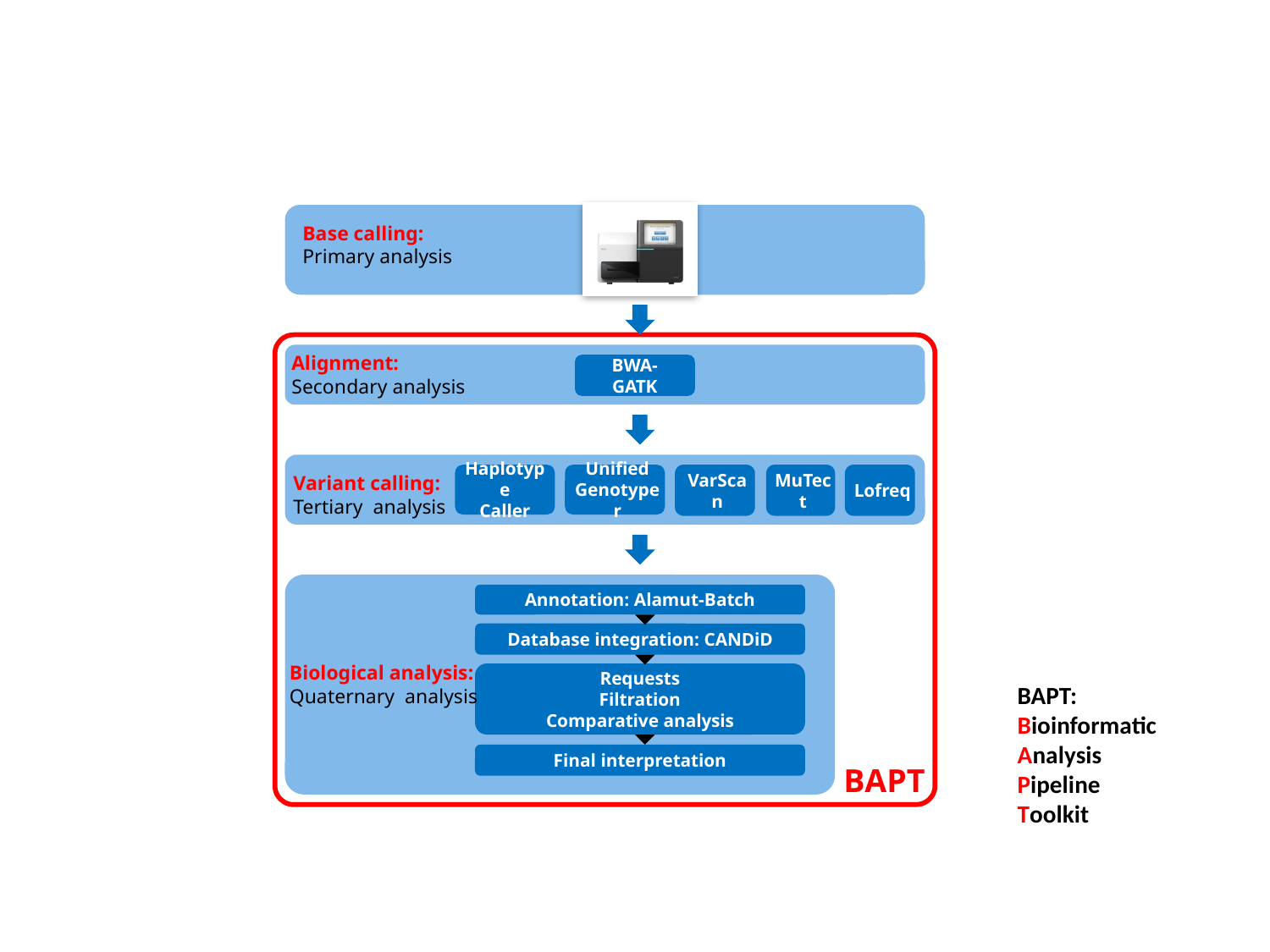

Base calling:
Primary analysis
Alignment:
Secondary analysis
BWA-GATK
Haplotype
Caller
Unified
Genotyper
VarScan
MuTect
Lofreq
Variant calling:
Tertiary analysis
Annotation: Alamut-Batch
Database integration: CANDiD
Biological analysis:
Quaternary analysis
Requests
Filtration
Comparative analysis
BAPT:
Bioinformatic
Analysis
Pipeline
Toolkit
Final interpretation
BAPT
